# Theta-gamma-coupling as predictor of working memory performance in young and elderly healthy people

**DOI:** 10.1186/s13041-024-01149-8

**Published:** 2024-10-16

**Authors:** Mohammed Abubaker, Wiam Al Qasem, Kateřina Pilátová, Petr Ježdík, Eugen Kvašňák

**Affiliations:** 1 Department of Medical Biophysics and Medical Informatics, Third Faculty of Medicine, Charles University in Prague, Prague, Czechia; 2 Department of Information and Communication Technology in Medicine, Faculty of Biomedical Engineering, Czech Technical University in Prague, Prague, Czechia; 3 Department of Circuit Theory, Faculty of Electrical Engineering, Czech Technical University in Prague, Prague, Czechia

**Keywords:** Transcranial alternating current stimulation (tACS), Theta-gamma coupling, Working memory (WM), Electroencephalography (EEG), Power spectral density (PSD)

## Abstract

The relationship between working memory (WM) and neuronal oscillations can be studied in detail using brain stimulation techniques, which provide a method for modulating these oscillations and thus influencing WM. The endogenous coupling between the amplitude of gamma oscillations and the phase of theta oscillations is crucial for cognitive control. Theta/gamma peak-coupled transcranial alternating current stimulation (TGCp-tACS) can modulate this coupling and thus influence WM performance. This study investigated the effects of TGCp-tACS on WM in older adults and compared their responses with those of younger participants from our previous work who underwent the same experimental design. Twenty-eight older subjects underwent both TGCp-tACS and sham stimulation sessions at least 72 h apart. Resting-state electroencephalography (EEG) was recorded before and after the interventions, and a WM task battery with five different WM tasks was performed during the interventions to assess various WM components. Outcomes measured included WM task performance (e.g., accuracy, reaction time (RT)) and changes in power spectral density (PSD) in different frequency bands. TGCp-tACS significantly decreased accuracy and RT on the 10- and 14-point Sternberg tasks and increased RT on the Digit Symbol Substitution Test in older adults. In contrast, younger participants showed a significant increase in accuracy only on the 14-item Sternberg task. Electrophysiological analysis revealed a decrease in delta and theta PSD and an increase in high gamma PSD in both younger and older participants after verum stimulation. In conclusion, theta-gamma coupling is essential for WM and modulation of this coupling affects WM performance. The effects of TGCp-tACS on WM vary with age due to natural brain changes. To better support older adults, the study suggests several strategies to improve cognitive function, including: Adjusting stimulation parameters, applying stimulation to two sites, conducting multiple sessions, and using brain imaging techniques for precise targeting.

## Introduction

Working memory (WM) is the ability to store and process information for short periods of time [1]. It is essential for advanced skills such as planning, mathematical ability and logical thinking [2]. There are various models for understanding WM [3–6], with Baddeley and Hitch’s model being widely accepted. According to this framework, WM consists of three core components: (1) the phonological loop, which is responsible for processing verbal information; (2) the central executive, which is responsible for updating WM representations, switching between task rules, and suppressing irrelevant responses; and (3) the visuospatial sketchpad, which is dedicated to processing visual and spatial information [3, 7, 8]. WM involves three primary processes: encoding, maintenance and processing of information, and retrieval of stored information [9, 10]. From a theoretical perspective, the terms “Short-term memory” (STM) and WM are used to illustrate how information is temporarily stored. STM refers to the ability to store information for a short time without actively processing it. The main difference between STM and WM lies in the aspect of information processing [11, 12].

Aging is a complex natural process that leads to varying degrees of cognitive decline [13]. Some seniors retain acceptable cognitive function, while others experience varying degrees of cognitive decline that can lead to pathological conditions [14]. This decline can significantly impair a person’s ability to live independently [15]. Therefore, researchers are continually exploring methods aimed at mitigating or even reversing both the physiological and pathological aspects of cognitive decline [16, 17]. The peak of cognitive abilities is reached at different ages [18–20]. For instance, abilities associated with fluid intelligence, such as STM, generally peak in early adulthood. Meanwhile, crystallized intelligence abilities such as vocabulary generally peak in middle age [18, 21]. However, recent findings complicate this dichotomy [19, 20]. For example, STM for names peaks at age 22, whereas STM for faces and quantity discrimination doesn’t peak until age 30 [19, 20]. There are different results with regard to WM [22–27]. Some researchers suggest that cognitive abilities peak around the age of 30 and then slowly decline, while others point to a sharp decline in cognitive function between the ages of 50 and 60 [26, 28–32]. Conversely, some argue for a gentler decline without precisely defining a specific age limit [27, 33–35]. Different types of information are processed by different temporal memory systems, with visuospatial WM declining more markedly with age than verbal WM [10, 22, 36–38]. There are many theories to explain the decline in WM observed in older people [39–42]. These include reduced storage resources indicating deficits in bottom-up processing [39], impairments in executive control [43], less efficient inhibitory processes [42], a general slowing of cognitive processing [41], an observable degradation of the neural substrate in the frontal lobe [40], difficulties in linking information to specific contexts leading to retrieval confusion [44], and reduced attentional resources. In addition, functional neuroimaging research has shown that activation of the left prefrontal cortex, an important region for WM processes, decreases with age [45, 46]. Deficits in WM have also been found in a number of neurological and psychiatric disorders, including schizophrenia, major depressive disorder (MDD), attention-deficit/hyperactivity disorder (ADHD), Alzheimer’s disease and bipolar affective disorder [47–50].

Neuronal oscillations or brain waves are rhythmic or repetitive patterns of neuronal activity in the central nervous system. They can be observed in different frequency ranges and are associated with different cognitive functions and states of consciousness [51–53], and are mainly categorized into five frequency ranges: Delta, theta, alpha, beta, and gamma. Neuronal oscillations play an important role in the connection and communication between brain regions [54]. They are characterized by a unique coupling property between different frequency bands, in particular cross-frequency coupling (CFC) [55]. Within large cortical networks, this coupling enables the interaction and synchronization of local and global processes [56]. CFC has been studied in humans and animals and involves the correlation between instantaneous frequency, phase and amplitude in different frequency bands [55, 57–60]. It has been associated with a wide range of disease states and cognitive functions [55, 60, 61]. A variety of CFC types have been identified, including: (1) Phase-amplitude coupling (PAC), a common type of CFC, shows how the phase of oscillations in a frequency band (beta, theta, etc.) and the amplitude of oscillations in another frequency band (usually gamma oscillations) can be synchronized; (2) Amplitude-amplitude coupling depicts the relationship between the amplitudes of oscillations in two different frequency bands; (3) phase-phase coupling characterizes the correlation between the phases of oscillations in different frequency band; and (4) phase-frequency coupling depicts the relationship of the phase of oscillations in one band and the frequency of oscillations in another band [57]. All brain oscillations contribute to WM processes, with theta and gamma oscillations being particularly emphasized. Theta oscillations are specifically associated with the encoding and retrieval of information and facilitate communication between different brain regions during WM tasks [62–64]. Gamma oscillations, on the other hand, are associated with the active maintenance of information during WM processes, which is necessary for integrating various features of an object or memory to form a coherent representation [65–67]. Research indicates an interaction between theta and gamma oscillations through PAC. This means that the amplitude of the gamma oscillations is modulated by the phase of the theta oscillations. It is assumed that this coupling plays a crucial role in the organization and coordination of neuronal activity during WM tasks [57, 58, 65, 68]. This interaction is often referred to as the theta-gamma neural code. Within this framework, two models have been proposed. The first model assumes that each gamma wave represents a single memory item [69, 70]. In contrast, the second model assumes that a single memory item is encoded by the entire gamma burst that occurs within the theta cycle [71, 72]. According to the first model, there is a finite number of gamma waves (memory items) that can fit into a given theta cycle, which explains why WM capacity is limited. Another explanation for the limited WM capacity is provided by the second model, which suggests that new gamma bursts are needed to refresh the memory content over time, with reactivation occurring after a few theta cycles [69–72].

The relationship between memory and oscillations can be investigated by manipulating neuronal oscillations to causally influence behavior [73]. Three main approaches can be used to achieve oscillatory entrainment, i.e. the modulation of brain oscillations: non-invasive electrical/magnetic stimulation [74, 75], invasive electrical stimulation [76, 77], and sensory entrainment [78, 79]. Transcranial alternating current stimulation (tACS) is a specialized form of non-invasive transcranial electrical stimulation (tES). tACS can be administered at frequencies based on naturally occurring oscillations and is primarily used as a modulator of cognitive abilities [80]. The effectiveness of stimulating ongoing oscillations is highest when the frequencies of the stimulus match the endogenous frequencies [81, 82]. tACS can be applied either online during cognitive tasks or offline immediately before or between tasks. The sustained brain activity post-stimulation, referred to as the “aftereffect” [83], suggests lasting changes in synaptic plasticity rather than mere entrainment per se [84]. Cognitive tES studies, often focus on the prefrontal cortex (PFC). Modulation of the dorsolateral PFC has been shown to improve attention, multitasking and memory and can also be used to treat psychiatric disorders [85–90]. Given the link between irregularities in cortical oscillations and various neuropsychiatric and cognitive disorders [54, 91], tACS is a promising approach for treating brain disorders and improving cognition [92, 93]. To achieve optimal stimulation, parameters such as location, intensity, frequency of stimulation need to be considered, but there is no standardized protocol yet.

The effects of tACS on WM are frequency-dependent, with theta and gamma oscillations being particularly relevant. They have mainly been studied in young, healthy adults. For effective modulation, it is crucial that certain brain regions, such as the prefrontal cortex, are addressed. However, the effectiveness of tACS varies according to individual differences, task-specific requirements and stimulation parameters, including frequency, intensity, duration and electrode placement. Research suggests that tACS can improve WM performance, however, results are conflicting and some studies report no or even negative effects [66, 94–102] and others. Posterior theta tACS, stimulation at two sites (e.g. fronto-parietal), and temporo-parietal gamma tACS have shown significant improvements [94–96, 98, 99, 101, 103–107]. Understanding the neurophysiological effects of tACS through methods such as electroencephalography (EEG) and magnetoencephalography (MEG) [87, 88, 107–111] is crucial for optimizing these protocols and treatments. Further research is needed to develop standardized tACS protocols to improve WM. One promising approach that has been explored in recent studies is peak-coupled theta/gamma tACS (TGCp-tACS) [108, 112]. This technique takes advantage of a natural process in the brain in which gamma bursts align with the peak of theta waves, an interaction that is critical for memory, attention and other cognitive functions [113]. TGCp-tACS enhances this natural coupling by synchronizing the gamma bursts with the theta peaks and can thus increase cognitive performance. In a study conducted by [108], TGCp-tACS was applied to the left frontal cortex in young, healthy subjects during a visuospatial WM task and showed improved WM performance, particularly at gamma frequencies between 80 and 100 Hz [108]. In support of these findings [112], used frequency-tuned TGCp-tACS during a modified Sternberg task and also observed improvements. Although limited, all studies on TGCp-tACS effects on WM in young participants have shown promising results [108, 112]. Further research is needed to confirm its efficacy across different WM components and age groups to assess its broader potential.

The effects of tACS on WM in older adults have not been studied as thoroughly as in younger adults [114–118]. In contrast to sham stimulation [114], found that tACS targeting the parietal alpha frequency increased target responses in a retro-cue task [115]. discovered that applying tACS with a beta frequency (20 Hz) over the parietal central region accelerated responses in a visual-tactile delayed match-to-sample task, while tACS with a gamma frequency (70 Hz) slowed them down. In addition [117], reported that fronto-temporal, in-phase, theta-tuned tACS enhanced local theta/gamma coupling (TGC) in older adults, which increased their accuracy in a change detection task. In another study [116], tACS with different stimulation parameters was applied for three or four consecutive days to improve verbal WM and long-term memory in older volunteers. Their results showed that theta-tACS applied over the inferior parietal lobe improved WM on the third and fourth day, with the effect lasting up to one month after stimulation. On the other hand [118], used personalized bifocal theta-tACS targeting the frontoparietal network and found improvements in motor sequence learning tasks with high WM load compared to sham stimulation, although tasks with low WM load did not show similar benefits. Details of the studies can be found in Table 1.

**Table 1:** Summary of studies investigating the effects of tACS on WM in older adults

**Study#1 (Reinhart and Nguyen, 2019)** [117]	
Description	• Theta-tACS was applied concurrently at two sites with synchronized phases. • Younger adults were only subjected to sham stimulation. • Older adults underwent both sham and active stimulation sessions. • Four experiments were conducted, each with different conditions: sham stimulation, fronto-temporal in-phase theta-tuned stimulation, unifocal frontal theta-tuned stimulation, and unifocal temporal theta-tuned stimulation. • Additionally, a non-tuned stimulation at 8 Hz targeting the fronto-temporal region and a theta-tuned HD-tACS with fronto-temporal anti-phase configuration were employed.
Age (range)	• (60–76 years).
Target cortex	• Left prefrontal and left temporal cortices.
Task(s)	• Change-detection paradigm. • Participants underwent 10 blocks of the task during stimulation followed by 20 blocks post-stimulation.
Outcomes	• Fronto-temporal in-phase theta-tuned tACS was found to augment local theta/gamma coupling in older adults. • Fronto-temporal in-phase theta-tuned tACS led to enhanced accuracy in a WM task. • The improvement in WM persisted for at least 50 min following 25 min of theta stimulation.
**Study#2 (Borghini et al., 2018)** [114]	
Description	• The study involved 50 participants, evenly distributed between younger and elderly adults. • In Experiment 1, both younger and older participants completed a WM retro-cueing task in a pre-stimulation session without tACS, serving as the baseline. • Experiment 2 focused on elderly participants performing the WM task across four sessions, each including either 4 Hz, 10 Hz, 35 Hz tACS, or sham stimulation. Sessions were spaced apart by at least 48 h, following a within-subject design. • Experiment 3 compared the effects of 10 Hz stimulation against sham stimulation in a task-irrelevant retro-cueing condition.
Age mean ± SD (range)	• Elderly: Mean age: 69.1 ± 4.5 years; (62–78 years). • Young adults: Mean age: 24.8 ± 4.3 years; (18–33 years).
Target cortex	• Bilateral parietal cortices.
Task(s)	• Retro-cue WM task
Outcomes	• The administration of alpha-tACS to the parietal region in elderly participants led to a higher rate of target responses compared to sham stimulation.
**Study#3** [116]	
Description	• The study involved 33 participants, including healthy younger and older adults. • The effects of 20 Hz beta and 70 Hz gamma-tACS on WM outcomes were investigated.
Age mean ± SD	• Young adults: 24.4 ± 3.1 years. • Older adults: 72.6 ± 4.6 years.
Target cortex	• Parieto-central region
Task(s)	• Visuo-tactile delayed match-to-sample task.
Outcomes	• 20 Hz (beta) tACS resulted in faster response times. • Conversely, 70 Hz (gamma) tACS led to delayed responses. • The acceleration effect of beta-tACS was particularly pronounced in older adults.
**Study #4** [119]	
Description	• The study included 20 healthy older adults. • Personalized theta-tACS was administered to bifocal fronto-parietal networks, compared to sham stimulation.
Age mean ± SD	• 69.6 ± 4.4 years
Target cortex	• Bifocal fronto-parietal networks.
Task(s)	• Participants engaged in sequence learning task • N-back task • Various WM loads
Outcomes	• Application of personalized theta tACS to the fronto-parietal networks enhanced performance, both in terms of accuracy and response time, only during motor sequence learning task with a high WM load. • The stimulation paradigm resulted in improved performance on the N-back task, particularly for the 2-back task, but did not significantly affect performance for the 1-back and 3-back tasks.
**Study#5** [116]	
Description	• Repetitive tACS spanning three or four days were designed to evaluate the potential enhancements in auditory-verbal WM and long-term memory among older adults. • Data from a cohort of 150 older participants were analysed. • Participants were randomly allocated to one of three neuromodulation groups in Experiments 1 and 2, or to two groups in Experiment 3. 1. Experiment 1 (day 1–4), interventions included Sham, left DLPFC gamma (60 Hz), and left IPL theta (4 Hz) stimulations. 2. Experiment 2 (day 1–4) involved Sham, left DLPFC theta (4 Hz), and left IPL gamma (60 Hz) stimulations. 3. Experiment 3 (day 1–3), participants received left DLPFC gamma (60 Hz) and left IPL theta (4 Hz) stimulations. • WM assessments were conducted on days 1 through 4, and one month after stimulation in Experiments 1 and 2, while in Experiment 3, assessments occurred on days 1 through 3.
Age (range)	• (65–88 years).
Target cortex	• Left DLPFC and left IPL
Task(s)	• Classic immediate free recall task
Outcomes	• Theta-tACS targeted the left IPL demonstrated a preference for enhancing WM on day 3, day 4, and 1-month after intervention, while gamma-tACS applied over left DLPFC exhibited a preference for enhancing long-term memory on days 2 through 4, and 1-month after intervention.

tACS: transcranial-alternating current stimulation; HD-tACS: high-definition tACS; WM: working memory; DLPFC: dorsolateral prefrontal cortex; IPL: inferior parietal lobe

tACS has demonstrated potential as a non-invasive and safe technique for modulating brain oscillations to causally influence cognition. Studies in younger adults have shown that modulation of TGC with tACS can improve certain aspects of WM. However, previous research has focused primarily on younger populations, leaving the effects of TGCp-tACS on various components of WM and the effects in older adults largely unexplored. The present study aimed to investigate the effects of TGC modulation through tACS on WM in cognitively intact older participants. This study is among the first to explore the impact of this specific stimulation protocol in older adults, considering both behavioral and neurophysiological outcomes. To enhance sensitivity and detect potential behavioral modulation, we utilized a set of five distinct tasks, each designed to measure specific components of WM, thereby extending our investigation across multiple aspects of WM. Additionally, electroencephalography (EEG) was utilized to monitor changes in brain oscillations, with a particular focus on TGCp-tACS-induced alterations in power spectral density (PSD) across different frequency bands. Furthermore, this study compared the results from older adults with those of younger participants from our previous research conducted under identical experimental conditions (Al Qasem W, Abubaker M, Pilátová K, Ježdík P, Kvašňák E. Improving working memory by electrical stimulation and cross-frequency coupling. Accepted for publication 2024). This comparison offers valuable insights into age-related differences in brain responses to stimulation, helping to guide the development of future interventions to prevent or mitigate cognitive decline in older populations. Below is an overview of the tasks employed in our study, synthesized from various sources in the existing literature. This summary provides insights into the methodologies and frameworks commonly used in similar research.

The Wisconsin Card Sorting Test (WCST) is a widely recognized test for assessing abstract thinking and cognitive flexibility that can be used in both clinical and research contexts [119, 120]. The test is flexible to use and allows for various adaptations, e.g. the comprehensive version with 128 cards, the WCST-64 variant using only the initial set of 64 cards, and the WCST-3 variant which is terminated after completion of the first three categories, etc [121, 122]. Smith-Seemiller and colleagues (1997) suggested that the shortened versions of the WCST (i.e., WCST-64 and WCST-3) produced results on cognitive assessments comparable to those of the full 128-card WCST [121]. The arrangement within the decks of cards can vary, from random sequences to structured rules designed to prevent the successive occurrence of certain types of cards [122–124]. The criteria for moving to a new category may depend on a certain number of correct responses or cards [122, 125–127]. The diversity of WCST applications makes it difficult to compare the results of different studies. Despite its widespread use, there is still confusion in the scientific literature about the most appropriate assessment methods and the interpretation of results as indicators of cognitive flexibility and abstract thinking. An important measure for assessing cognitive flexibility is the number of perseverative errors, which reflect participants’ inability to change their response strategies after rule changes [120, 127, 128], i.e., continuing to apply an outdated rule despite feedback to the contrary. In our study, we used the shortened WCST-3 version. The specific details of the task utilized in this study are thoroughly described in the “Methods 2.5”. The primary outcomes measured in this task are the number of perseverative errors and the reaction time (RT). Reaction time refers to the interval between the presentation of a stimulus and the participant’s response to it. Measuring RT allows us to assess the speed of cognitive processing, which are critical indicators of performance efficiency and cognitive function.

The Digit Symbol Substitution Task (DSST) serves as an assessment tool to evaluate a person cognitive abilities, focusing on processing speed, attention and executive functions [129]. In the paper-and-pencil version of the task, participants are initially presented with a key containing digits paired with specific symbols. They are then shown additional rows of digits and instructed to quickly fill in the corresponding symbol for each digit within a given time frame [130]. The DSST is available in several formats, from traditional paper-and-pencil tests to digital versions [129, 131, 132], making it a versatile tool for assessing cognitive function. It captures different cognitive domains and challenges the notion that it only measures processing speed. The DSST covers key elements of executive functions such as inhibition, cognitive flexibility and updating [133]. This complexity arises from the requirement for participants to not only process information quickly, but also to deal effectively with and adapt to changing information. An inherent challenge in evaluating executive functions through tasks like the DSST is the issue of “task impurity,” which highlights the challenge in isolating and measuring specific executive functions due to their overlap with other cognitive processes [8, 133, 134]. In summary, performance on the DSST reflects not only processing speed, but also a broader range of cognitive and executive abilities [135]. The detailed description of the tasks used in this study can be found in the “Methods 2.5” section. The main outcomes measured in this study are accuracy and RT.

The Sternberg Task investigates how participants store, maintain, and retrieve items for short period of time. This task encompasses three sequential stages: encoding, maintenance and retrieval. During the encoding stage, participants are briefly presented with verbal/visual information (usually letters) [136–139]. In the maintenance stage, the verbal information is kept in mind through subvocal rehearsal, and in the retrieval stage, the stored information is utilized to formulate a response [136]. Building on Baddeley’s 2003 theory of the phonological loop, it’s understood that visual input, like the letters in the Sternberg task, is converted into a phonological format for storage. In contrast, auditory input is stored directly without the need for conversion [140, 141]. Thus, the Sternberg task, whether in its verbal or visual form, can assess the function of the phonological loop. In our study, we used the Sternberg task with different levels of difficulty. The details of the task utilized in this study can be found in the “Methods 2.5” section. The primary outcomes measured in this study include accuracy and RT, providing a comprehensive evaluation of performance metrics.

The Flanker task is a psychological test to assess attention and the ability to prevent cognitive interference [142, 143]. It assesses a person’s ability to focus on a central stimulus while ignoring distracting stimuli that flank it [144]. During the task, individuals are asked to quickly and accurately recognize the direction in which the central arrow is pointing while ignoring the surrounding distracting stimuli. These distractions can be congruent, i.e. they match the direction of the central arrow, incongruent, i.e. they do not match the direction of the central arrow, or neutral, i.e. they do not contain a directional cue that could influence the decision about the direction of the central arrow [145, 146]. The main outcomes measured are: Accuracy and reaction time (RT). A detailed description of the tasks used in this study is provided in the “Methods 2.5” section. The primary metrics evaluated in this study include accuracy and RT.

The visuospatial WM task [147, 148] assesses a person’s ability to temporarily store and process visual and spatial information. In this task, participants are presented with an array of colored squares on a screen. After a short pause, a second array appears and participants must decide whether it matches the first one. Some versions have distractions, such as different colored rectangles, which can be used to measure how well participants can ignore irrelevant information. The detailed procedures for the task used in this study are described in section “Method 2.5”. The primary measures evaluated in this study include accuracy and RT.

## Materials and methods

### Participants

The study involved 28 right-handed, non-color-blind adults aged 50 years and older (14 women, age 63 ± 9.37). Each participant gave written informed consent prior to participation. All participants had normal or corrected-to-normal vision and no psychiatric or neurological disorders, metal implants, implanted electronic devices, brain injuries, or medications that affect the nervous system. None of the subjects had contraindications to tACS and were unfamiliar with the tasks and stimulation procedures. Cognitive function was assessed prior to the experiment by a psychologist using the Montreal Cognitive Assessment (MoCA) to ensure that cognitive abilities were intact. The study complied with the ethical standards of the Declaration of Helsinki and was approved by the Ethics Committee of the Third Faculty of Medicine of Charles University in Prague.

### Study design

**Fig. 1:**
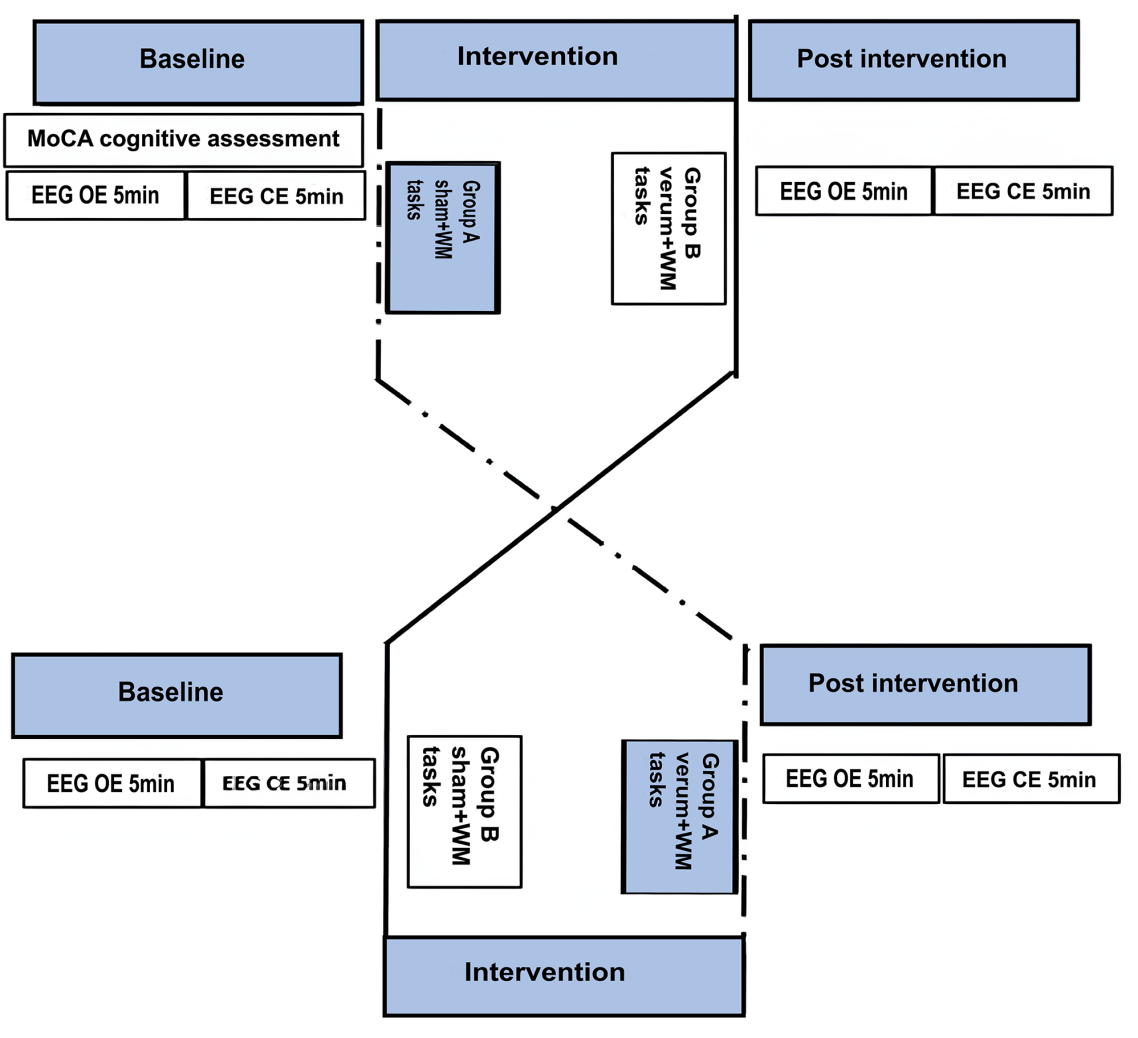
Visual representation of the experimental setup. EEG: electroencephalography; OE: open-eyes; CE: closed-eyes

The experimental protocol for this study was derived from procedures described in our previous work (Al Qasem W, Abubaker M, Pilátová K, Ježdík P, Kvašňák E. Improving working memory by electrical stimulation and cross-frequency coupling. Accepted for publication 2024),but was specifically modified to include comprehensive cognitive assessments for seniors prior to the experiments. Participants attended two separate sessions — sham and verum stimulation — each separated by at least 72 h to minimize potential carryover. The order of the sessions was counterbalanced between the participants. An introductory session was held at the beginning to familiarize participants with the laboratory environment and experimental procedures. Each session began with a 5-minute resting-state EEG recording with eyes open, followed by a 5-minute recording with eyes closed. Participants then received either sham or verum stimulation for 20 min while performing tasks from a WM battery, and then received a 5-minute resting-state EEG recording with eyes open, followed by a 5-minute recording with eyes closed. A visual representation of the experimental setup can be found in Fig. 1.

### Equipment and experimental procedure

In the study, Starstim^®^ tES-EEG technology from Neuroelectrics-Barcelona with NIC v2.0.11.7 software was used for both EEG recording and electrical stimulation. Ag/AgCl electrodes were placed at 20 standard locations on the scalp according to the international 10–20 system. The key regions for recording were frontal, temporal, central, parietal and occipital (Fp1, Fp2, Fz, F3, F4, F7, F8, Cz, C3, C4, T7, T8, Pz, P3, P4, P7, P8, Oz, O1, and O2). Electrical stimulation was performed with five NG-Pistim electrodes (1 cm radius, π cm² contact area) filled with conductive EEG gel. The central electrode was positioned over F3, with return electrodes at Fp1, Fz, C3 and F7. The stimulation signal had a sampling rate of 1 MHz, an analog-to-digital precision of 14 bits (≈ 0.5 µA), and the electrode impedance was kept below 10 kOhm. Signal quality was ensured by continuous monitoring. Participants were instructed to avoid activities that could cause EEG artifacts, such as blinking, eye movements, swallowing, chewing, and talking. All experiments were performed in a laboratory free of sound and electromagnetic signals.

### Stimulation protocol

An alternating current with a peak to baseline value of 1 mA was administered for 20 min, including a 10-second fade-in and a 10-second fade-out period. This protocol combined a 6 Hz theta wave with 0.6 mA peak to baseline with synchronized 80 Hz gamma bursts with 0.4 mA peak to baseline, each lasting 50 ms at the peak of the theta wave. Synchronization was controlled and verified using special hardware and an oscilloscope. In the sham condition, stimulation was automatically terminated after 30 s.

### Assessment of WM components

Participants were seated 50 cm away from a 24-inch monitor (resolution 2560 × 1440, 60 Hz) and WM assessments were performed using E-prime software. After an initial standardized training phase, participants completed the WM test battery within 20 min under either TGCp-tACS or sham conditions, with the order of tasks pseudorandomized and counterbalanced. Performance data, including RT, accuracy, and number of perseverative errors, were recorded. The test battery comprised the following subtests:

#### Visuospatial WM task

Participants observed a series of red and blue rectangles at different angles on the screen for 500 ms. They memorized the orientation of the red rectangles on one side of the screen, which was indicated by a directional arrow. After a short retention period (900 ms), a test screen appeared and participants indicated whether the orientation matched the memorized configuration by pressing “1” for a match and “2” for a mismatch. The task involved two (2-stimulus) or four (4-stimulus) red rectangles (targets) and two to six blue rectangles (distractors) and was repeated in nine cycles for a total of 72 trials.

#### Sternberg task

Participants memorized a series of letters in black and ignored letters in green. After seeing a mixed list of black and green letters, with each letter displayed for 1000 milliseconds (ms). Following this, a new series of red letters appeared, each displayed for 3000 ms. During this time, participants had to indicate whether the red letter matched any letter from the memorized set by pressing “1” for a match and “2” for a mismatch. This task comprised four cycles with two blocks each: the first block with eight letters to be memorized and 14 letters to be evaluated (14-item) and the second block with another eight letters to be memorized and 10 letters to be evaluated (10-item), which is less cognitively demanding.

#### Flanker task

Participants identified the direction of a central arrow surrounded by congruent, incongruent or neutral symbols using the keyboard (“1” for left, “2” for right). The task consisted of 141 trials under three conditions: congruent (e.g., <<<<<), incongruent (e.g., <<><<), and neutral (e.g., 
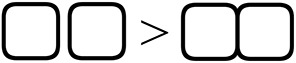
). Conditions. Each trial was displayed for 1000 milliseconds (ms), and participants had to respond within a 1-minute timeframe.

#### DSST

Participants learned digit-symbol pairs (e.g., 10 = “=”). The symbols then flashed on the screen and participants had to match each symbol to the correct digit. They had to form as many pairs as possible within 180 s.

#### WCST

Participants sorted cards by color, number or symbol, with each sorting trial lasting for 5000 ms. The rules for categorization changed every 14 cards in 42 trials, so participants had to dynamically adapt their strategies. Immediate feedback was given for each sorting decision.

### Processing the EEG data

The EEG data were preprocessed using a standardized MATLAB script based on the EEGLab Toolbox [149]. The raw EEG data were resampled to 256 Hz, and direct current (DC) shifts were removed to ensure baseline stability. An FIR high-pass filter (0.5 Hz cutoff) was applied to minimize low-frequency noise. Artifact subspace reconstruction (ASR) was used to clean the raw data. The raw data time series were visually inspected to remove any additional artifacts, followed by the application of average referencing. Independent component analysis (ICA) was used to identify and remove artifacts from eye blinks, heart signals, and other non-brain activity. The cleaned data was transformed into the frequency domain using the Fast Fourier Transform (FFT) with a Hamming window, a window size of 256 points, and an overlap of 50%. The power spectra obtained from these windows were averaged and then converted to a logarithmic scale, facilitating the effective analysis of power spectral density (PSD) across the delta, theta, alpha, beta, and gamma bands.

### Statistical methods

PSD were compared using paired t-tests with false discovery rate (FDR) corrections for multiple comparisons using FieldTrip implemented in the EEGLAB environment [149, 150], maintaining statistical significance at p-values below 0.05. Behavioral data were analyzed using Generalized Linear Mixed-Effects Model (GLMM) fitted by Penalized Likelihood (PL) that account for repeated measures and include fixed effects (e.g., age, task conditions) as well as random effects to account for individual differences. This approach ensured robust statistical conclusions by taking into account the interdependence of multiple measurements from the same participants under different conditions.

## Results

### Peak-coupled theta/gamma tACS reduces accuracy but improves reaction time in the sternberg task

The analysis of the results of the Sternberg task in 10-item and 14-item conditions showed different TGCp-tACS effects on accuracy and RT. In the 10-item condition, verum stimulation significantly reduced accuracy by 3.6% (*p* = 0.01, 95% CI: -6.4% to -0.8%) and improved RT by 47 ms (*p* = 0.01, 95% CI: -81.8 to -13.1 ms). Age also had a negative effect on accuracy, with a decrease of 0.34% per year (*p* < 0.001, 95% CI: -0.5% to -0.17%), but had no significant effect on RT (*p* = 0.7).

In the 14-item condition, TGCp-tACS significantly decreased accuracy by 2.7% (*p* = 0.04, 95% CI: -5.4% to -0.2%) and improved RT by 65 ms (*p* < 0.001, 95% CI: -93.2 to -37.5 ms). Age had a negative effect on accuracy with a decrease of 0.21% per year (*p* = 0.006, 95% CI: -0.37% to -0.06%), but had no significant effect on RT (*p* = 0.535). Overall, TGCp-tACS did not improve accuracy, but actually reduced it, but it did improve RT. Aging had a negative effect on accuracy, but no significant effect on RT.

Peak-coupled theta/gamma tACS slows reaction time in the Digit Symbol Substitution Task without significantly affecting accuracy.

**Fig. 2:**
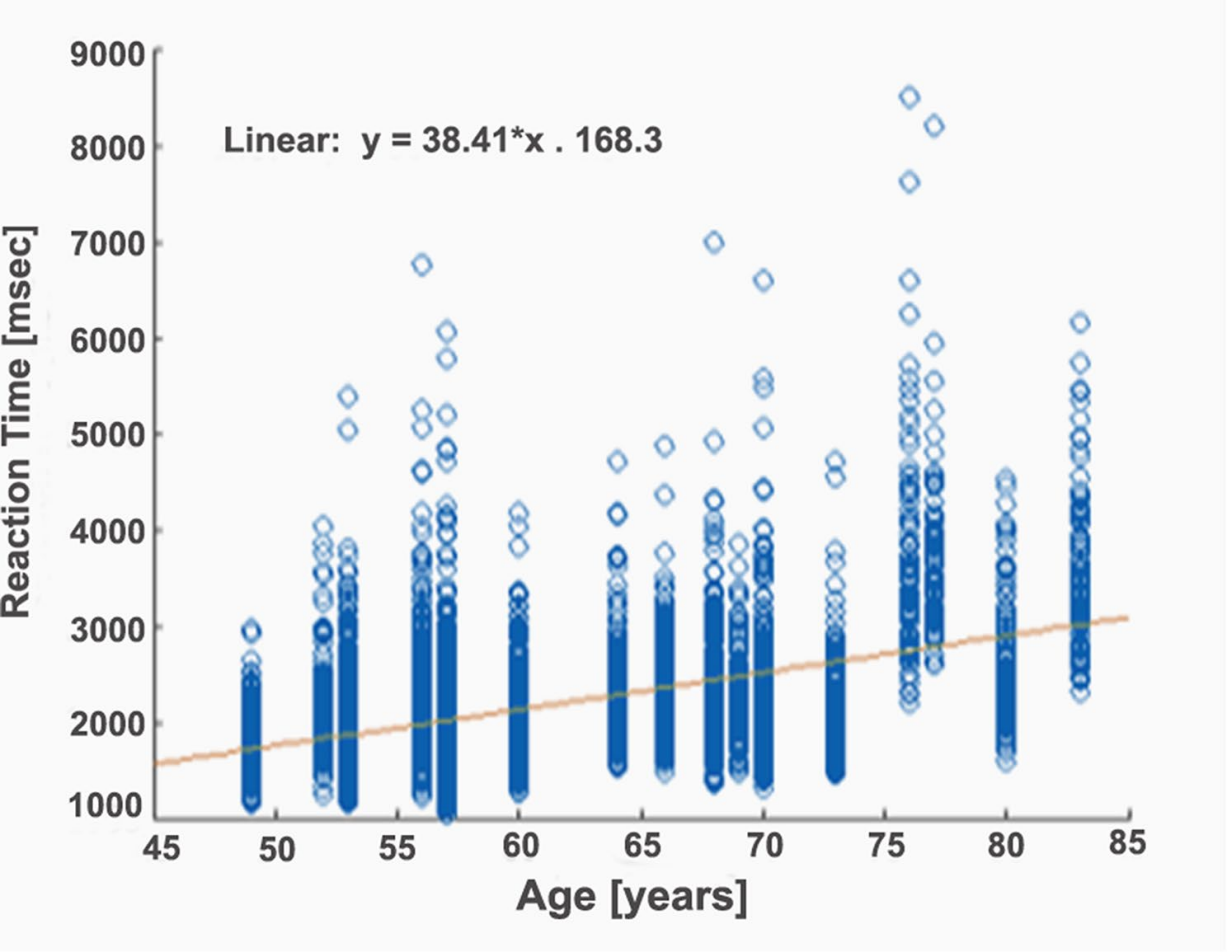
Age-Dependent Increase in RT during the DSST. The figure illustrates the relationship between participant age and reaction time in the DSST. As age increases, reaction times also increase, demonstrating a clear age-dependent trend. DSST: Digit Symbol Substitution Task

For DSST, the analysis showed that TGCp-tACS reduced accuracy by 0.27% (*p* = 0.10, 95% CI: -0.60–0.06%). For RT, TGCp-tACS significantly increased RT by an average of 35.59 ms (*p* = 0.03, 95% CI: 3.01 to 68.17 ms), indicating slower RT compared to sham sessions. In addition, age had a significant effect on RT, with each additional year increasing RT by an average of 40.23 ms (*p* < 0.001, 95% CI: 26.21 to 54.25 ms). Overall, while TGCp-tACS did not significantly improve accuracy, it unexpectedly slowed RT, and RT became progressively slower with increasing age Fig. 2 illustrates the relationship between participant age and RT in DSST.

No significant performance effect of peak-coupled theta/gamma tACS in the Flanker TaskAnalysis of the effects of TGCp-tACS on the Flanker task showed no significant improvements in RT under the congruent, neutral and incongruent conditions. Verum stimulation caused a change in RT of 1.10 ms (*p* = 0.74, 95% CI: -5.34 to 7.54) for the neutral condition, -0.92 ms (*p* = 0.78, 95% CI: -7.32 to 5.48) for the congruent condition and 4.86 ms (*p* = 0.23, 95% CI: -3.01 to 12.74) for the incongruent condition, all indicating no significant effect. Conversely, age had a significant effect on RT in all conditions, with increases of 2.47 ms (*p* = 0.02, 95% CI: 0.37 to 4.57), 2.54 ms (*p* = 0.02, 95% CI: 0.44 to 4.64) and 2.78 ms (*p* = 0.01, 95% CI: 0.58 to 4.98) per year for neutral, congruent and incongruent conditions, respectively. Due to the exceptionally low error rate among participants, accuracy was not a meaningful measure for performance evaluation, so we rely solely on RT for performance assessment. Overall, TGCp-tACS did not improve performance in the flanker task, while aging negatively affected RT.

### Peak-coupled theta/gamma tACS shows no significant changes in reaction time or perseverative errors in the wisconsin card sorting task

Analysis of the effect of TGCp-tACS on the WCST revealed no significant improvements in the number of perseverative errors or RT. There was no significant difference in the number of perseverative errors between verum and sham sessions, with a change of 0.002 (*p* = 0.9, 95% CI: -0.026 to 0.03). For RT, verum stimulation decreased RT by 22.69 ms (*p* = 0.38, 95% CI: -73.1 to 27.7 ms). However, age had a significant effect on RT, with each additional year increasing RT by 16.5 ms (*p* = 0.001, 95% CI: 6.3 to 26.6 ms). Overall, TGCp-tACS did not reduce the number of perseverative errors or RT, while age was associated with slower RT.

No significant impact of peak-coupled theta/gamma tACS on Visuospatial Working Memory Task performanceAnalysis of the visuospatial WM task results in two conditions (2 stimuli and 4 stimuli) showed that TGCp-tACS did not improve accuracy significantly in the 2 stimuli condition with a change of 3.53% (*p* = 0.07, 95% CI: -0.30%, 7.35%). The RT showed a non-significant increase of 7.56 ms (*p* = 0.60, 95% CI: -20.51, 35.63). In the 4 stimuli condition, accuracy decreased by -3.80% (*p* = 0.09, 95% CI: -8.24%, 0.63%) due to verum stimulation, and RT showed a non-significant increase of 6.55 ms (*p* = 0.66, 95% CI: -22.57, 35.68). Age had a significant negative effect on accuracy in both conditions. In the 2-stimulus condition, each year of age was associated with a decrease in accuracy of 0.77% (*p* = 0.0003, 95% CI: -1.18%, -0.36%) and a non-significant decrease in RT of 3.50 ms (*p* = 0.33, 95% CI: -10.51, 3.52). For the 4-stimulus condition, each year of age was associated with a decrease in accuracy of 0.41% (*p* = 0.03, 95% CI: -0.78%, -0.04%) and a non-significant decrease in RT of 5.43 ms (*p* = 0.14, 95% CI: -12.60, 1.73). Overall, TGCp-tACS did not significantly improve accuracy or RT on Visuospatial WM task in the older participants.

Peak-coupled theta/gamma tACS impairs specific aspects of working memory in elderly participants but enhances them in young participants.

**Fig. 3:**
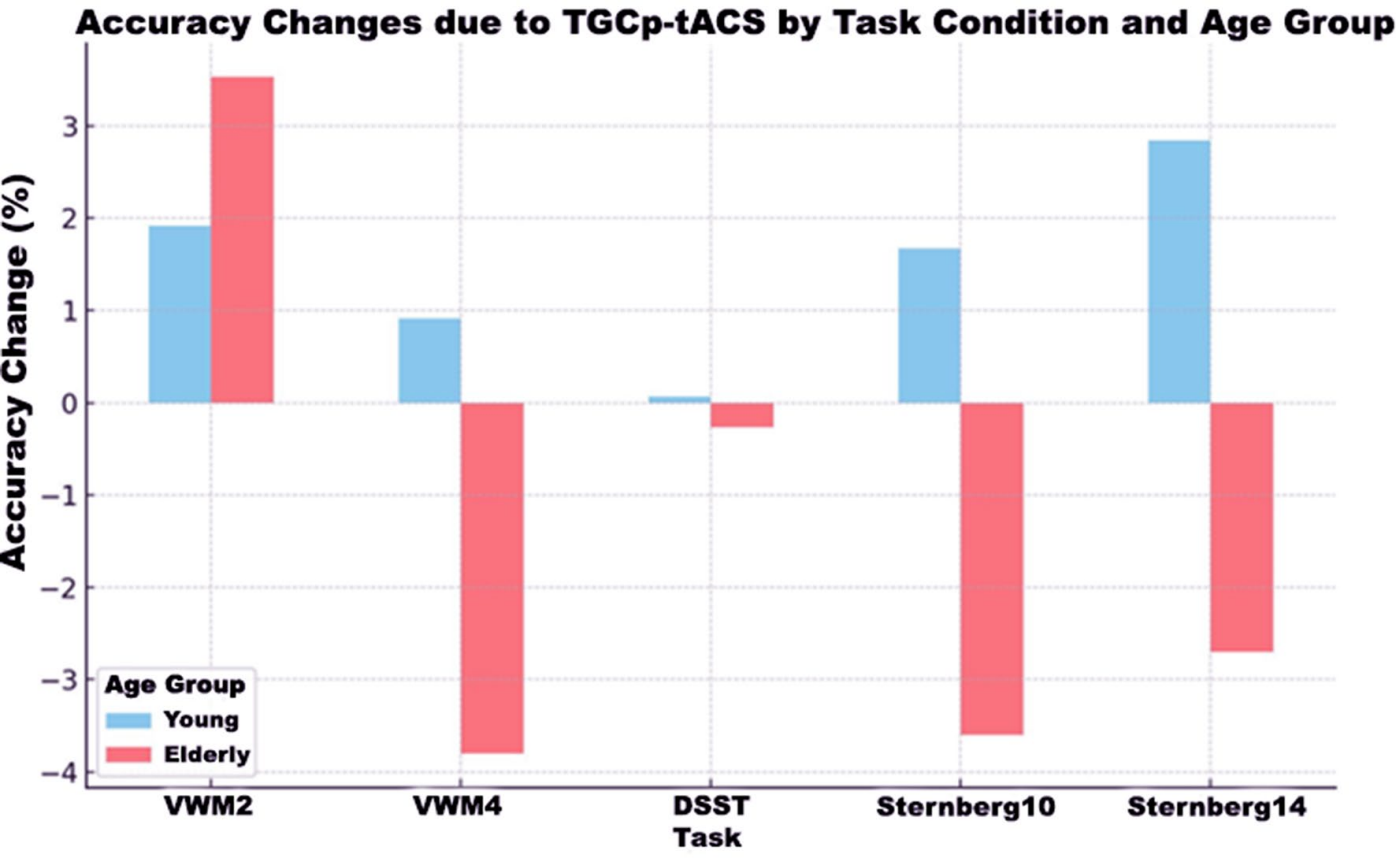
Percentage Changes in Accuracy for Young and Older Participants in Various Cognitive Tasks due to TGCp-tACS. The bar chart illustrates the percentage changes in accuracy for young and older participants in different cognitive tasks due to TGCp-tACS. The changes in accuracy are shown in sky blue for young participants and in salmon for older participants. While most changes are not statistically significant, significant improvements are observed in the Sternberg 14-item condition for young participants and in both the Sternberg 10-item and 14-item conditions for older participants. TGCp-tACS: theta/gamma peak-coupled-transcranial-alternating current stimulation; VWM2: Visuospatial working memory (2-stimulus); VWM4: Visuospatial working memory (4-stimulus); DSST: Digit Symbol Substitiution task; Sternberg10: Sternberg task (10-item); Sternberg14: Sternberg task (14-item)

The results for the young participants are drawn from our previous work (Al Qasem W, Abubaker M, Pilátová K, Ježdík P, Kvašňák E. Improving working memory by electrical stimulation and cross-frequency coupling. Accepted for publication 2024). In the Sternberg task, TGCp-tACS significantly improved accuracy in the 14-item condition for young participants without significantly affecting RT. Conversely, TGCp-tACS significantly decreased both accuracy and RT in the 10-item and 14-item conditions for older participants. In the DSST, TGCp-tACS showed no significant effects on accuracy or RT for young participants, whereas in older participants, TGCp-tACS caused a slight decrease in accuracy and a statistically significant increase in RT, with age contributing significantly to this increase. In the Flanker task, TGCp-tACS had no significant effect on RT in any condition for either age group, although age significantly increased RT in older participants. In the WCST, TGCp-tACS had no significant effect on the number of perseverative errors or RT in either age group, although age significantly increased RT in older participants. In the visuospatial WM task, TGCp-tACS had no significant effect on accuracy or RT in the two- or four-stimulus conditions for either age group. However, age significantly decreased accuracy in older participants without significantly affecting RT. Overall, although not significant in most tasks, TGCp-tACS tended to improve accuracy on all cognitive tasks for young participants, contrasting with older participants, for whom stimulation caused a decrease in accuracy on most tasks. Aging generally had a negative effect on accuracy and increased RT in older participants. These results are detailed in Table 2, which outlines the changes in RT, accuracy, and the number of perseverative errors under different task conditions and age groups due to verum stimulation. Additionally, Fig. 3 graphically depicts the changes in accuracy by task and age group due to TGCp-tACS.

**Table 2:** Detailed comparison of changes in accuracy (%), Reaction times (ms), and number of perseverative errors across various cognitive Tasks for young and elderly participants

Task	Metric	Young volunteers			Senior volunteers		
		Value	*p*-value	95%CI	Value	*p*-value	95%CI
**Visuospatial WM (2 stimuli)**	Accuracy Change (%)	1.92	0.22	-1.18–5.02%	3.53	0.07	-0.30–7.35%
	RT Change (ms)	5.07	0.59	-13.44 to 23.58 ms	7.56	0.60	-20.51 to 35.63 ms
**Visuospatial WM (4 stimuli)**	Accuracy Change (%)	0.91	0.64	-2.90–4.72%	-3.8	0.09	-8.24–0.63%
	RT Change (ms)	17.42	0.07	-1.70 to 36.54 ms	6.55	0.66	-22.57 to 35.68 ms
**DSST**	Accuracy Change (%)	0.07	0.72	-0.31–0.45%	-0.27	0.10	-0.60–0.06%
	RT Change (ms)	-18.18	0.07	-37.86 to 1.50 ms	35.59*	0.03	3.01 to 68.17 ms
**Sternberg (10-item)**	Accuracy Change (%)	1.67	0.13	-0.49–3.82%	-3.6*	0.01	-6.4% to -0.8%
	RT Change (ms)	25.20	0.15	-9.03 to 59.43 ms	-47.0*	0.01	-81.8 to -13.1 ms
**Sternberg (14-item)**	Accuracy Change (%)	2.84*	0.01	0.63–5.06%	-2.7*	0.04	-5.4% to -0.2%
	RT Change (ms)	-18.61	0.22	-48.18 to 10.96 ms	-65.0*	0.001	-93.2 to -37.5 ms
**Flanker** **(Neutral)**	RT Change (ms)	-3.69	0.19	-9.15 to 1.76 ms	1.1	0.74	-5.34 to 7.54 ms
**Flanker (Congruent)**	RT Change (ms)	-0.36	0.90	-5.76 to 5.05ms	-0.92	0.78	-7.32 to 5.48 ms
**Flanker (Incongruent)**	RT Change (ms)	0.81	0.82	-6.19 to 7.82 ms	4.86	0.23	-3.01 to 12.74 ms
**WCST**	Number of Perseverative Errors changes	-0.01	0.50	-0.03 to 0.02	0.002	0.90	-0.026 to 0.03
	RT Change (ms)	-23.70	0.20	-59.76 to 12.37 ms	-22.69	0.38	-73.1 to 27.7 ms

* indicates p-value < 0.05 showing a significant difference.CI: confidence interval; RT: reaction time; Visuospatial WM: Visuospatial working memory, DSST: Digit Symbol Substitution task; WCST: Wisconsin Card Sorting task

**Fig. 4:**
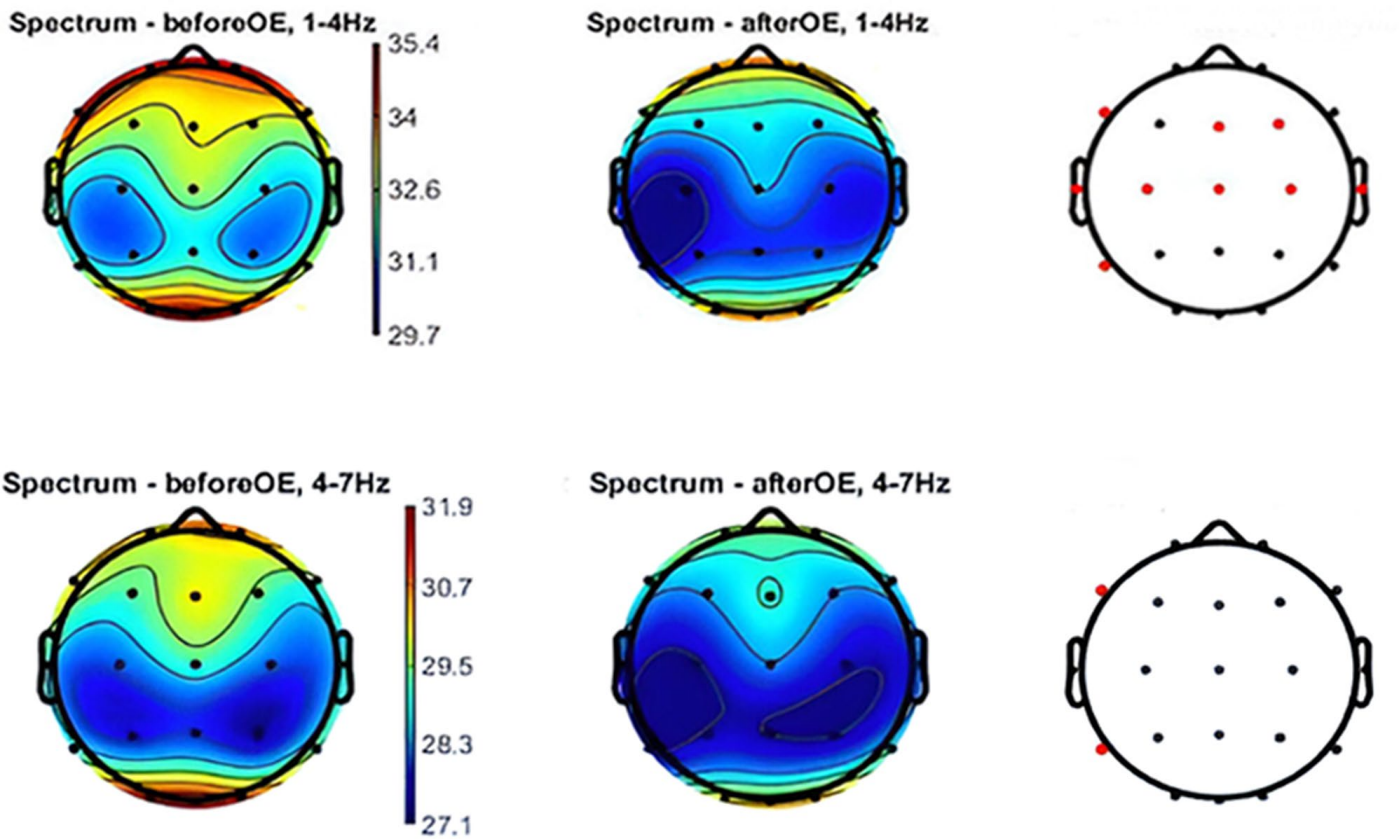
2D Topographic Maps of PSD Distribution in Theta and Delta Ranges. Sham condition-OE the figure displays 2D topographic maps illustrating the PSD distribution across various electrodes in the theta and delta ranges. Statistically significant differences observed before and after specific conditions are indicated by red dots. Statistical analysis was performed using paired t-tests with FDR correction for multiple comparisons (*p* < 0.05) utilizing the Fieldtrip toolbox. OE: open-eyes; CE: closed-eyes; Hz: hertz; PSD: Power spectral density; FDR: false discovery rate

Peak-coupled theta/gamma tACS induces a decrease in delta and theta power spectral density along with an increase in high-gamma power spectral density**Sham condition**: Eyes-open (after vs. before): There was a widespread decrease in delta and theta PSD that was statistically significant. The PSD of the other frequency bands remained relatively unchanged, with a negligible increase in the gamma and beta PSD. Figure 4 Displays 2D topographic maps of PSD distribution across various electrodes in the theta and delta ranges, highlighting statistically significant differences observed before and after specific conditions. With eyes-closed (after vs. before): Waiting 5 min before performing EEG recordings and closing the eyes led to a partial normalization of brain activity, potentially reducing the visibility of the effects of the task on certain EEG frequency bands.

#### Verum condition

**Fig. 5:**
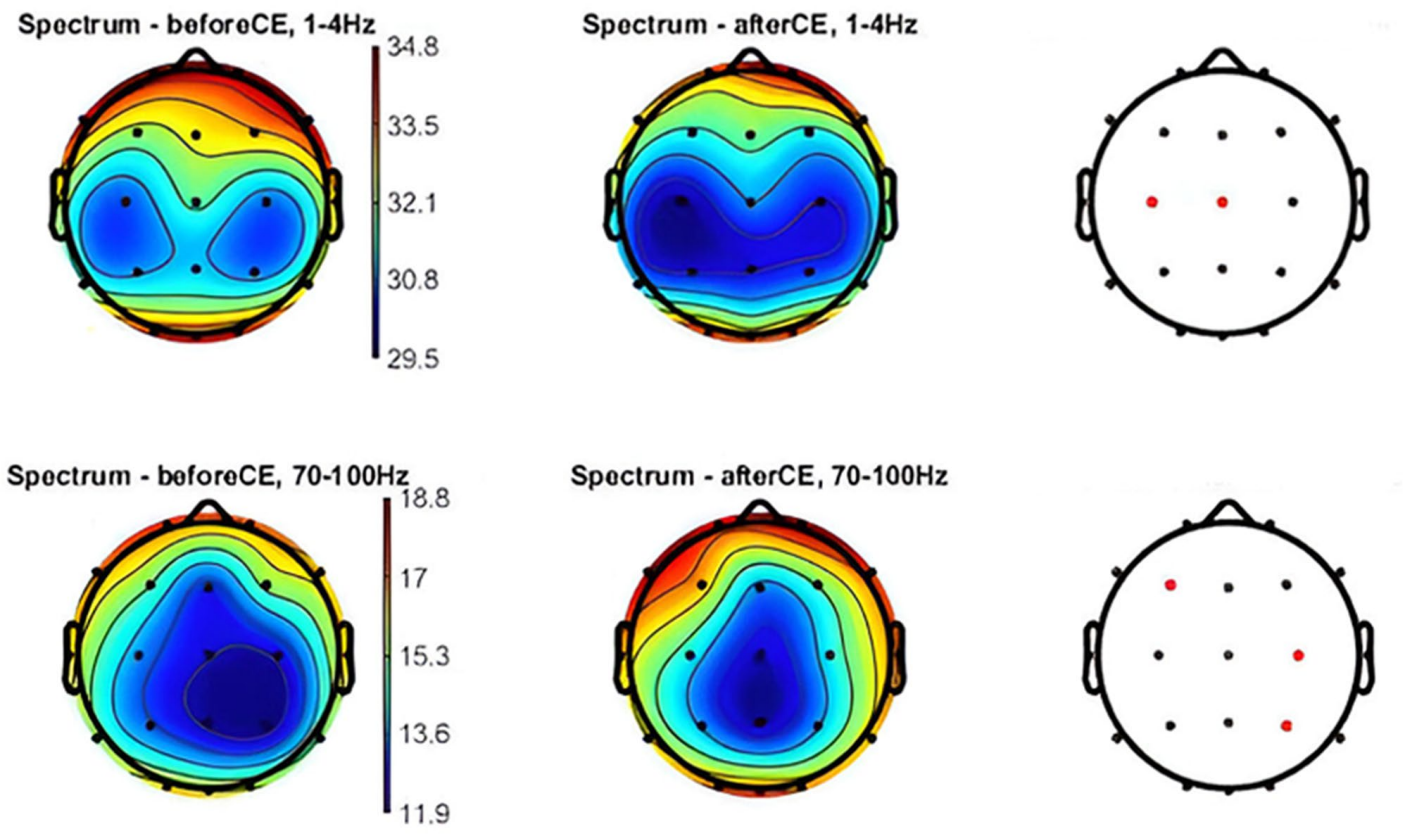
2D Topographic Maps of PSD Distribution in delta and high-gamma Ranges. Verum condition–CE the figure displays 2D topographic maps illustrating the PSD distribution across various electrodes in the delta and high-gamma. Statistically significant differences observed before and after specific conditions are indicated by red dots. Statistical analysis was performed using paired t-tests with FDR correction for multiple comparisons (*p* < 0.05) utilizing the Fieldtrip toolbox. OE: open-eyes; CE: closed-eyes; Hz: hertz; PSD: Power spectral density; FDR: false discovery rate

Eyes-open (after vs. before): Analysis revealed a slight global decrease in delta and theta PSD, which was not statistically significant, while PSD of other frequency bands showed minimal changes, with eyes-closed (after vs. before): The results showed a widespread decrease in delta PSD, which was statistically significant at the Cz and C3 positions. Theta PSD also showed a widespread decrease, but this was not statistically significant. The high gamma power showed a remarkable increase in the left frontal and right posterior and central regions, which was statistically significant. For more details refer to Fig. 5.

Peak-coupled theta/gamma tACS reduces low-frequency power spectral density and enhances high-gamma power spectral density in both young and elderly participants.

The changes in PSD across different brain regions following sham and verum stimulations are somewhat similar in both age groups. The results for young participants are derived from our previous work (Al Qasem W, Abubaker M, Pilátová K, Ježdík P, Kvašňák E. Improving working memory by electrical stimulation and cross-frequency coupling. Accepted for publication 2024). Table 3 describes the changes in PSD across electrodes under different conditions and age groups.

**Table 3:**
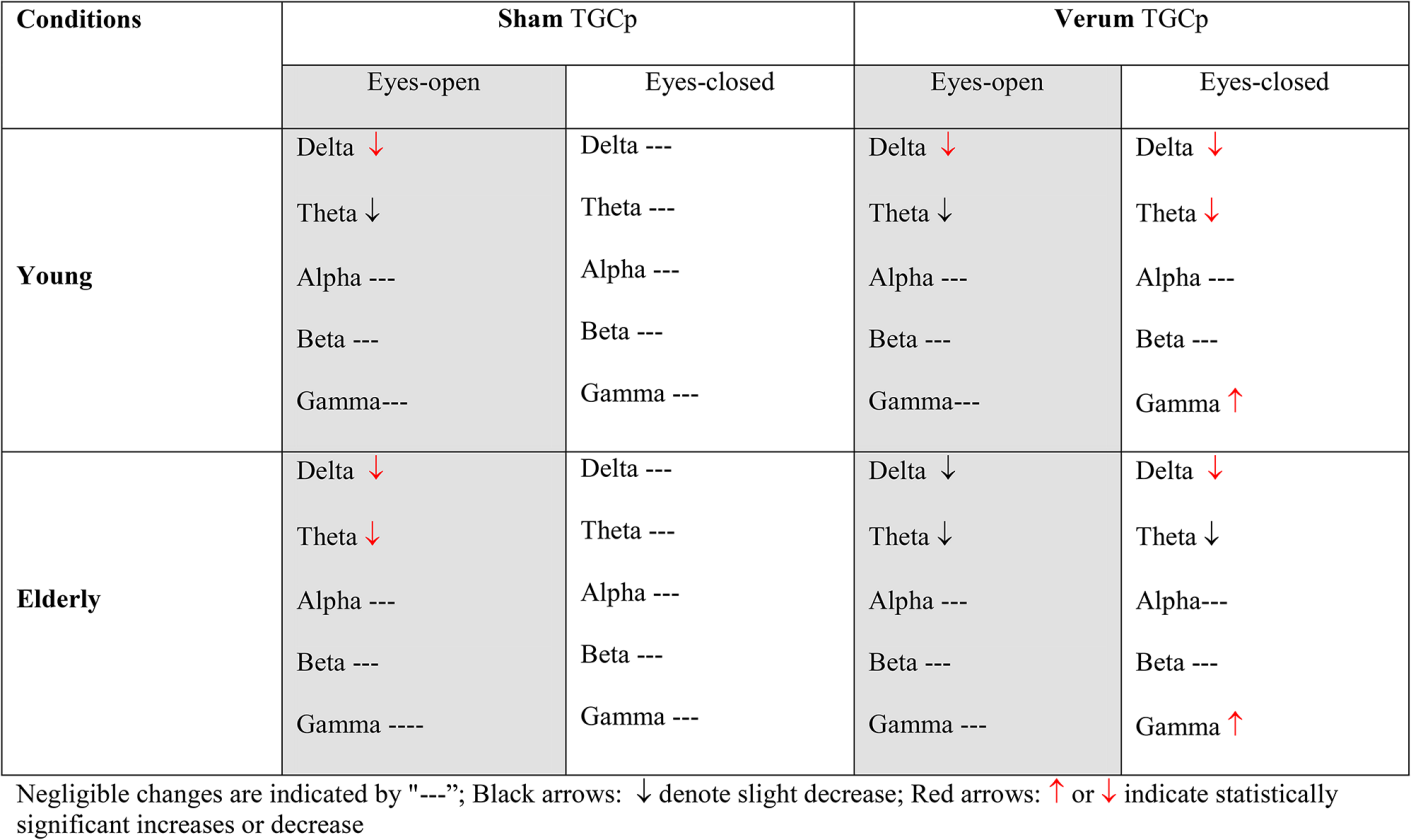
Changes in PSD across electrodes under various conditions and age groups

## Discussion

The relationship between WM and oscillations can be explored by manipulating neuronal oscillations to causally influence behavior through brain stimulation. In this study, we investigated the effects of TGCp-tACS on WM in older adults and compared the results with those of younger participants. By modulating endogenous theta/gamma coupling, TGCp-tACS can causally influence WM, underscoring the crucial role of this interaction in WM processes. Each participant underwent both TGCp-tACS and sham stimulation sessions at least 72 h apart so that each person could serve as their own control. Resting-state EEG was recorded before and after the interventions (sham or verum stimulation), and participants completed a WM task battery during the intervention. This groundbreaking study investigates the potential of TGCp-tACS to improve WM in older adults, inspired by encouraging results in younger participants that showed improvements in specific components of WM [108, 112]. Our findings showed that TGCp-tACS resulted in a statistically significant decrease in accuracy and RT on the 10- and 14-item Sternberg tasks and a significant increase in RT on the DSST in older adults. In contrast, younger participants showed a statistically significant increase in accuracy only on the 14-item Sternberg task. Electrophysiological observations revealed that in the eyes-open condition with sham stimulation, there was a significant decrease in theta and delta PSD, especially in older participants, and a negligible increase in beta and gamma PSD, indicating that the brain remains in an alert state immediately after task completion due to higher cognitive load. In the eyes-closed condition with sham stimulation, brain activity returned to baseline across all frequency bands at least five minutes post-task completion in both age group. In verum stimulation with eyes open, young participants experienced a significant decrease in delta PSD and a non-significant decrease in theta PSD, while older participants showed non-significant decreases in both delta and theta PSD. This condition, measured immediately after task completion, suggests a complex interaction between sensory inputs, task-related changes in brain dynamics, and stimulation effects, which may obscure clear observations of the stimulation effects. In the eyes-closed condition with verum stimulation, younger participants demonstrated a statistically significant decrease in delta and theta PSD and an increase in high gamma PSD, particularly at the F3 position. Older participants exhibited a statistically significant decrease in delta power and a significant increase in high gamma power in the F3, right posterior, and central areas. Compared to the eyes-closed condition with sham stimulation, where brain dynamics return to baseline, the observed effects in the eyes-closed condition with verum stimulation can be attributed to the stimulation. These findings suggest that measuring brain activity with eyes closed, at least five minutes after task completion, provides clearer insights into the effects of TGCp-tACS. This approach reduces visual input and minimizes transient neuronal changes associated with task processing, allowing for a more accurate assessment of the stimulation effects.

It is hypothesized that tACS produces its aftereffects through spike-timing-dependent plasticity (STDP), a process in which the timing of neuronal spikes influences the strength of synaptic connections. These effects can manifest as either long-term potentiation (LTP) or long-term depression (LTD). LTP is associated with a strengthening of synaptic connections, while LTD is associated with their weakening [151]. It has been proposed that STDP may be the underlying mechanism of the observed power changes in EEG frequency bands after stimulation [152]. administered tACS at the individual alpha frequency to the occipital cortex, and they found that alpha power increased significantly after stimulation compared to sham control. They suggested that when the stimulation frequencies that are at or slightly lower than the resonance (or endogenous) frequency would lead to synaptic strengthening (i.e., LTP); If the stimulation frequency is higher than the endogenous frequency would lead to a weakening of the synapse (i.e., LTD). Based on these results, the decreased in theta PSD and increase in high-gamma PSD observed in our study could be explained as follows: the fixed theta frequency 6 Hz might be significantly different from the individual`s theta frequency leading to synaptic weakening (LTD), while the fixed gamma frequency of 80 Hz is much closer to the endogenous gamma frequency, leading to synaptic strengthening (LTP). Furthermore, TGCp-tACS might have broad effects that particularly impact the delta band, leading to a decrease in delta PSD. The decrease in theta activity due to LTD could indirectly reduce delta PSD because of the strong interconnections and coupling between these frequencies [153, 154]. This relationship could explain the observed decrease in delta PSD.

Our understanding of how tES affects the brain comes largely from studies with younger participants, making it difficult to apply these findings directly to older adults [155–157]. While some effects of tES in older adults are similar to those in younger individuals [158–160] many studies show clear age-related differences [161–163]. In our study, we compared the effects of TGCp-tACS on WM outcomes between younger and older participants. We found that although both groups experienced similar changes in PSD due to the stimulation, their behavioral outcomes differed significantly. This discrepancy can be explained by the physiological and structural differences between younger and older brains, which may affect how TGCp-tACS influences cognitive function. As we age, our brains undergo various changes such as altered brain excitability, connectivity, reduced white matter integrity, cortical shrinkage and dysregulated neuronal plasticity due to decreased Gamma-aminobutyric acid (GABA) levels [158, 159, 164–169]. For example, older adults rely heavily on theta oscillations for cognitive processes [63, 170] If TGCp-tACS disrupts oscillations, it could have a negative impact on their performance. The stimulation frequencies used in this study may not be optimal for older adults, as evidenced by the decreased theta PSD post-stimulation. Older adults typically have lower theta peak frequencies. The discrepancy between their natural theta frequency and the applied 6 Hz frequency could disrupt their slower theta rhythm and effectively “speed up” their theta frequency. This shift could reduce the number of WM items they can process in a theta cycle and thus impair their WM capacity “introduction section”. This phenomenon is particularly critical because older adults are highly dependent on theta oscillations for WM processes. This dependency could explain the negative effect of TGCp-tACS at fixed theta 6 Hz and fixed gamma 80 Hz on the performance of WM tasks in the elderly compared to younger adults. In addition, differences in cortical thickness could lead to inter-individual and age-dependent variability in the electric fields induced by tES [171, 172], which in turn leads to different responses to TGCp-tACS [173]. For example, in individuals with thicker cortex, adjustments of stimulation parameters, such as intensity or electrode placement, might be required to effectively influence brain activity, as the electric currents have to cross more brain tissue. Although older participants generally have thinner cortical thickness than younger participants, the variability in cortical thickness is greater in older people [174–176]. These differences in cortical thickness could partly explain the different results in WM task outcomes. In addition, older adults often exhibit a natural decline in cognitive flexibility and processing speed, making it difficult for them to adapt to the new cognitive strategies required in the experiment [177]. Furthermore, it has been suggested that during cognitive tasks, older adults with poorer performance show increased activation in the right dorsolateral and rostrolateral prefrontal cortex compared to younger individuals [178] This may highlight the potential benefit of dual-site stimulation in elderly individuals, as targeting multiple brain regions simultaneously could help compensate for age-related cognitive decline.

The cognitive load of multiple WM tasks in a short period of time can be overwhelming for seniors, leading to increased guessing and difficulty in maintaining concentration, which in turn impairs performance. Environmental and contextual factors also play an important role. Older people may be more sensitive to the task environment, e.g., the comfort of the task environment or familiarity with the procedures. Anxiety or stress when performing cognitive tasks may affect older adults differently and potentially impair their performance during sessions [179]. While TGCp-tACS could improve task accuracy and speed up RT, it could also disrupt the precise neural networks required for high accuracy, especially in complex cognitive tasks. The range of responses — from improvement to no effect to deterioration in cognitive function — highlights the need for careful adjustment of stimulation parameters to maximize benefit.

### Future direction and limitation

This study has some limitations due to the use of non-personalized stimulation parameters and the complexity of the tasks, which may have influenced the evaluation of the results. In addition, the fact that participants had to complete numerous tasks in a limited time frame may have influenced the results.

### Strategies to improve the TGCp-tACS effectiveness on WM:

• Performing dual-site stimulation: Simultaneous application of TGCp-tACS to different brain regions (i.e., in particular the frontal and parietal regions) involved in WM processes may achieve a more effective result.

• Adapting WM tasks for older people: Adapting the complexity of WM tasks to the abilities of older participants may help to accurately assess the effects of TGCp-tACS on WM components without overtaxing them.

• Reducing task load per session: Limiting the number of WM tasks in a single session may prevent physical and mental fatigue in older adults and ensure that participants’ performance reflects their actual memory abilities rather than their endurance.

• Increasing session frequency: Previous research [180, 181] suggests that increasing the number of tACS sessions, e.g., one session per day for five consecutive days per week for four weeks, can significantly improve response to stimulation. This approach may lead to cumulative and long-lasting effects through mechanisms that promote neuroplasticity, which is particularly beneficial for older adults.

• Combining tACS with cognitive training: Integrating tACS with cognitive training or other therapeutic interventions may lead to greater cognitive improvements [181].

• Integration of tACS with electric field distribution modeling: By using advanced imaging techniques, such as Magnetic Resonance Imaging (MRI), to map the individual anatomy of the brain, a personalized model of the electric field distribution can be created. This method can overcome challenges arising from varying cortical thickness and other factors that influence the efficacy of TGCp-tACS [182].

• Multimodal imaging techniques: Combining multimodal imaging techniques (e.g., functional MRI (fMRI), Positron Emission Tomography (PET)) with TGCp-tACS can provide detailed insights into the brain’s response to stimulation. This approach allows for refined electrode placement and stimulation parameters to target specific neuronal pathways involved in the WM process [183].

## Conclusion

The study emphasizes the importance of theta-gamma coupling in WM and shows how modulation of this coupling pattern affects WM outcomes. A single TGCp-tACS session targeting the left frontal cortex affected WM outcomes in both young and older volunteers, resulting in similar changes in PSD but different behavioral outcomes. While younger volunteers generally showed better accuracy, older adults showed lower accuracy on most tasks, with a notable effect on the Sternberg task, which assesses the phonological component of WM. These differences are likely due to natural age-related brain changes. To better help older people, the study suggests several strategies to improve cognitive function: Adjusting stimulation parameters, especially in the lower gamma range, and adjusting individual theta frequency. This can be achieved by using EEG recordings either before stimulation or through a closed-loop protocol, applying stimulation at two sites, performing multiple sessions, and using brain imaging techniques, such as fMRI, for precise targeting.

## Data Availability

The datasets used and/or analyzed during the current study are available from the corresponding author on reasonable request.

## Abbreviations

ADHD: Attention-deficit/hyperactivity disorder
ASR: Artifact subspace reconstruction
CFC: Cross-frequency coupling
CE: Closed-eyes
DC: Direct current
DLPFC: Dorsolateral prefrontal cortex
DSST: Digit Symbol Substitution Task
EEG: Electroencephalography
FDR: False discovery rate
FFT: Fast Fourier transform
fMRI: Functional MRI
GLMM: Generalized Linear Mixed-Effects Model
HD-tACS: High-definition tACS
Hz: Hertz
ICA: Independent component analysis
IPL: Inferior parietal lobe
LTP: Long-term potentiation
LTD: Long-term depression
MDD: Major depressive disorder
MEG: Magnetoencephalography
MoCA: Montreal Cognitive Assessment
MRI: Magnetic Resonance Imaging
OE: Open-eyes
PAC: Phase-amplitude coupling
PET: Positron Emission Tomography
PL: Penalized Likelihood
PFC: Prefrontal cortex
PSD: Power spectral density
RT: Reaction time
STDP: Spike-timing-dependent plasticity
STM: Short-term memory
tACS: Transcranial alternating current stimulation
tES: Transcranial electrical stimulation
TGC: Theta/gamma coupling
TGCp-tACS: Peak-coupled theta/gamma tACS
WCST: Wisconsin Card Sorting Test
WM: Working memory
